# Is Buying and Drinking Zero and Low Alcohol Beer a Higher Socio-Economic Phenomenon? Analysis of British Survey Data, 2015–2018 and Household Purchase Data 2015–2020

**DOI:** 10.3390/ijerph181910347

**Published:** 2021-09-30

**Authors:** Peter Anderson, Amy O’Donnell, Daša Kokole, Eva Jané Llopis, Eileen Kaner

**Affiliations:** 1 Population Health Sciences Institute, Newcastle University, Baddiley-Clark Building, Newcastle NE2 4AX, UK; amy.odonnell@newcastle.ac.uk (A.O.); Eileen.kaner@newcastle.ac.uk (E.K.); 2 Department of Health Promotion, CAPHRI Care and Public Health Research Institute, Maastricht University, 6211 LK Maastricht, The Netherlands; d.kokole@maastrichtuniversity.nl (D.K.); eva.jane@esade.edu (E.J.L.); 3 Institute for Mental Health Policy Research, Centre for Addiction and Mental Health, Toronto, ON M5T 1R8, Canada; 4 ESADE Business School, Ramon Llull University, 08034 Barcelona, Spain

**Keywords:** no and low alcohol beer, social determinants, household purchases

## Abstract

Zero and low alcohol products, particularly beer, are gaining consideration as a method to reduce consumption of ethanol. We do not know if this approach is likely to increase or decrease health inequalities. The aim of the study was to determine if the purchase and consumption of zero and low alcohol beers differs by demographic and socio-economic characteristics of consumers. Based on British household purchase data from 79,411 households and on British survey data of more than 104,635 adult (18+) respondents, we estimated the likelihood of buying and drinking zero (ABV = 0.0%) and low alcohol (ABV > 0.0% and ≤ 3.5%) beer by a range of socio-demographic characteristics. We found that buying and consuming zero alcohol beer is much more likely to occur in younger age groups, in more affluent households, and in those with higher social grades, with gaps in buying zero alcohol beer between households in higher and lower social grades widening between 2015 and 2020. Buying and drinking low alcohol beer had less consistent relationships with socio-demographic characteristics, but was strongly driven by households that normally buy and drink the most alcohol. Common to many health-related behaviours, it seems that it is the more affluent that lead the way in choosing zero or low alcohol products. Whilst the increased availability of zero and low alcohol products might be a useful tool to reduce overall ethanol consumption in the more socially advantageous part of society, it may be less beneficial for the rest of the population. Other evidence-based alcohol policy measures that lessen health inequalities, need to go hand-in-hand with those promoting the uptake of zero and low alcohol beer.

## 1. Introduction

Alcohol use is a leading risk factor for ill-health and premature death [1,2]. The World Health Organization (WHO) has set a global target to reduce the harmful use of alcohol by 10% between 2010 and 2025 [3]. The WHO SAFER initiative calls on governments at all levels to: (I) Strengthen restrictions on alcohol availability; (II) Advance and enforce drink driving counter measures; (III) Facilitate access to screening, brief interventions and treatment; (IV) Enforce bans or comprehensive restrictions on alcohol advertising, sponsorship, and promotion; and (V) Raise prices on alcohol through excise taxes and pricing policies [4]. However, as stressed in WHO’s global alcohol strategy, there is also potential for the alcohol industry to contribute to these efforts by addressing the composition of its products [5], by, for example, reducing the amount of ethanol they contain [6]. In recent years, this appears to be gaining consideration amongst policy makers as well as in global alcohol markets [7,8]. Although not without public health advocates’ concern related to product descriptors and marketing [9], in its consultation document, ‘Advancing our health: prevention in the 2020s’ the UK Government made a commitment to work with the drinks industry to “deliver a significant increase in the availability of alcohol-free and low-alcohol products by 2025” [10].

Previously, we have shown that the introduction of new lower strength beers resulted in British households purchasing fewer grams of ethanol overall [11]. From 2015 to 2018, the introduction of 46 new zero or low alcohol (defined as 3.5% Alcohol by Volume (ABV) or less) beers was associated with reductions in purchases of grams of ethanol across all beer products and across all alcohol products [11]. Despite several evidence reviews concerning zero and low alcohol beer specifically [12,13], there remains a dearth of studies investigating which factors influence the actual purchasing and consumption of these products. Qualitative evidence suggests that choosing specific lower strength products tends to be driven by previous consumption of higher strength products from the same brand [14]. Other studies indicate that health and wellbeing issues, price differentials, and overall decreases in the social stigma associated with drinking alcohol-free beverages may also drive decisions to buy and consume zero and low alcohol beers [15].

Although there is some limited grey literature on the topic [16], we do not know if the purchase and consumption of zero and low alcohol beers is influenced by demographic and socio-economic characteristics of consumers, and whether it occurs across all socio-economic groups, or it is restricted to more advantaged socio-economic groups. Using British survey data and British household purchase data, the aim of the study is to determine if the likelihood of buying and drinking zero alcohol beer and low alcohol beer differs by gender, age, social grade group, income, level of area-based residential deprivation, and region of Great Britain.

## 2. Methods

### 2.1. Study Design

We undertook binary logistic cross-sectional regression analyses to estimate the likelihood of drinking or buying zero alcohol beer or low alcohol beer by socio-demographic characteristics of respondents using online survey data and household purchase data.

### 2.2. Data Sources

We used two data sources: (1) household purchase data that capture numbers of adults in the household, age of the main shopper, social grade group of the household, household income, level of area-based residential deprivation, and region of Great Britain (as household purchase data captures data for the whole household, there is no specific data by gender); and (2) survey data that captures for each respondent, sex, age, social grade, level of area-based residential deprivation, and region of Great Britain. These are described in detail below.

### 2.3. Household Purchase Data

We used data from Kantar Worldpanel’s (KWP) household shopping panel for the years 2015 to 2020, the years for which we had data. The shopping panel comprises approximately 30,000 British households at any one time, recruited via stratified sampling, with targets set for region, household size, age of main shopper, and occupational group. Households record all off-trade purchases from all store types, including Internet shopping, brought back into the home using barcode scanners. To be included in KWP’s final datasets, households must meet quality control criteria (meeting thresholds for data recording and purchasing volume or spend (based on household size) every four weeks), with some 90–95% of households included [17]. In our sample, out of 79,411 households providing purchase data on 5.02 million separate alcohol purchases, 11,598 households (14.6%) did not provide household income data. Missing income data were imputed using monotonic multiple imputation [18]. There was no other missing data.

The data included the truncated postcode of each household (up to first four characters, two letters and two numbers). Alcohol purchases are recorded daily. For each individual purchase, the data includes the type and volume of the purchase using 19 drink categories, the brand, and the alcohol by volume (ABV). The volume purchased was combined with ABV to calculate grams of alcohol purchased.

We grouped households into: (I) four groups of the age of the main shopper (18–34; 35–44; 45–64; and 65+ years); (II) four occupation-based social grade groups (AB (‘highest’), C1, C2, DE (‘lowest’))with AB including higher and intermediate managerial, administrative or professional workers, C1 supervisory or clerical and junior managerial, administrative or professional workers, C2 skilled manual workers, and DE semiskilled and unskilled manual workers, state pensioners, casual and lowest grade workers, and unemployed with state benefits only based on the National Readership Survey [19]; (III) four similar sized household income groups (£0–8.75 k; >£8.75–15 k; >£15–22.5 k; and >£22.5 k per adult per household per year); (IV) four similar sized groups of the number of grams of all alcohol regularly purchased (>0–7; >7–21; >21–70; and >70 g of alcohol purchased per adult per household per week, averaged over the total number of days between first and last recorded day of an alcohol purchase); and, (V) four similar sized groups of area-based residential deprivation ranging from 1 (most deprived) to 4 (least deprived) based on multiple indices of ranking of residential deprivation aggregated at truncated postcode level with the lowest number of ranking the most deprived, and the highest number of ranking the least deprived for each of England [20], Scotland [21], and Wales [22]. The multiple indices include measures of income, employment, education, health, crime, access to housing, and environmental quality.

We classified three beer groups: zero-alcohol beer with an ABV = 0.0%; low-alcohol beer with an ABV > 0.0% and ≤3.5%; and all other beer with an ABV > 3.5%.

### 2.4. Alcovision Survey Data

We used data from Kantar WorldPanel (KWP) Alcovision survey for the years 2015–2018, the years for which we had data. Alcovision is an ongoing cross-sectional online timeline follow-back (TLFB) diary survey of the previous week’s alcohol consumption, with an annual sample of approximately 30,000 individuals aged 18+ years in Great Britain. In the diary-based approach, participants provide detailed data on the consumption of alcohol during the previous seven days for each day. Each drink by named brand is recorded, with the volume consumed and whether the consumed drink was purchased on-trade (pubs, bars, restaurants, etc.) or off-trade (supermarkets, shops, etc.). Participants complete the survey only once, without repeated surveys. Quota samples based on age, sex, social grade group based on occupation, and geographic region are drawn from Kantar’s managed access panel. Invitations to participate are sent out on set dates and timed such that completion dates of the survey occurs every month, and each day of the year is represented in the data. Weights based on age-sex groups, social class, and geographical region are constructed using UK census data to ensure representativeness of British adults, with 18–34-year-olds oversampled.

Drink diaries were completed by 104,635 respondents (53,409 men and 51,226 women) during the four years from 2015 to 2018, with an average of 512 diaries per week, (SD = 173), a rate which remained stable over the four-year period (F = 0.544, *p* = 0.462).

The data includes the truncated postcode of each respondent (up to first four characters, two letters and two numbers). The number of drinks consumed by brand is recorded separately for on-and off-trade, with information given on serving sizes in millilitres (mL). Drinks are categorized into 19 categories. For beer products, the brand-specific ABVs from household purchase data were used [11]. There was no missing data.

We grouped respondents into (I) male or female; (II) four age groups (18–34; 35–44; 45–64; and 65+ years); (III) the same four occupation-based social grade groups as for the household purchase data (AB (‘highest’), C1, C2, DE (‘lowest’)), based on the National Readership Survey [18]; (IV) four similar sized groups of the number of grams of all alcohol consumed during the previous week (≤100; >100 to ≤200; >200 to ≤350; and, >350); and, (V) four similar sized groups of area-based residential deprivation ranging from 1 (most deprived) to 4 (least deprived) based on multiple indices of residential deprivation aggregated at truncated postcode level for each of England [20], Scotland [21], and Wales [22].

We classified three beer groups: zero-alcohol beer with an ABV = 0.0%; low-alcohol beer with an ABV > 0.0% and ≤3.5%; and all other beer with an ABV > 3.5%.

### 2.5. Statistical Analyses

We undertook separate binary logistic regression analyses for the purchase data and the survey data, using both univariate (individual models for each independent variable) and multivariate (with all independent variables in the model) analyses. For the purchase data, the dependent variables were binary variables at the level of the household, had or had not ever purchased any of zero or low alcohol beer on any day of purchase of any alcohol product. For the survey data, the dependent variables were binary variables at the level of the respondent, had or had not consumed any of zero or low alcohol beer during the previous week. For the purchase data, we added date of first alcohol purchase and number of days between first and last purchase of any alcohol as covariates. For the survey data, we added date of completing the survey as a covariate.

For all analyses, we report odds ratios with 95% confidence intervals. All analyses were performed with SPSSv26 (IBM, Armonk, NY, USA) [23].

## 3. Results

### 3.1. Household Purchase Data

**Figure 1:**
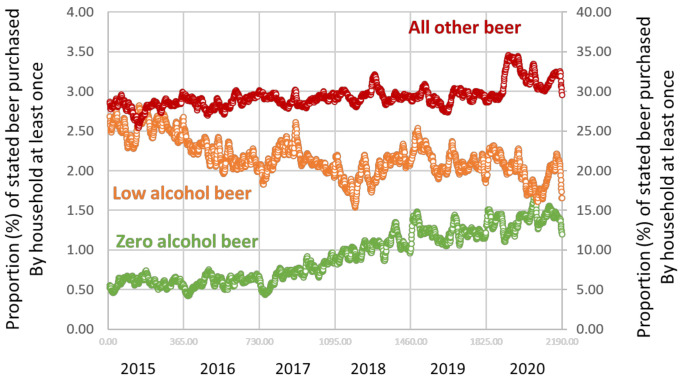
Proportion (%) of households that reported at least one purchase of zero alcohol beer (green, left vertical axis), low alcohol beer (orange, left vertical axis) and all other beer (red, right vertical axis) for any day that a household made an alcohol purchase by study day, 2015 to 2020. Data points: daily.

Based on the household purchase data, for any day that a household made an alcohol purchase, 0.92% (95% CI = 0.91 to 0.94) of households reported at least one purchase of zero alcohol beer, 2.17% (95% CI = 2.16 to 2.18) of low alcohol beer and 29.32% (95% CI = 29.26 to 29.38) of all other beer, Figure 1 (please note that the left vertical axis is for low and zero alcohol beer; and, the right vertical axis is for all other beer). The proportions of purchase of zero alcohol beer increased over time, whilst the proportions of low alcohol beer decreased over time. The proportion of all other beer showed a gradual increase over time, with an upwards blip during 2020 that coincided with the COVID-19 lockdown [24].

Table 1 gives the odds ratios (95% confidence intervals) for a household purchasing at least one zero alcohol beer and at least one low alcohol beer by household characteristics.

Households that were more likely to purchase zero alcohol beer were heavier purchasers of alcohol overall, and of beer with an ABV > 3.5%, were of younger age groups (highest odds for 35–44 year olds) and were relatively affluent (i.e., from higher household incomes, ‘higher’ social grades, and less deprived residential areas). There was no consistent pattern by region of Great Britain.

Households that were more likely to purchase low alcohol beer were also heavier purchasers of alcohol overall, and of beer with an ABV > 3.5%, and were more likely to be middle-aged (45–64 years old) than from a younger or older age group. There was no consistent pattern by household income, social grade, level of residential deprivation, or region of Great Britain.

**Figure 2:**
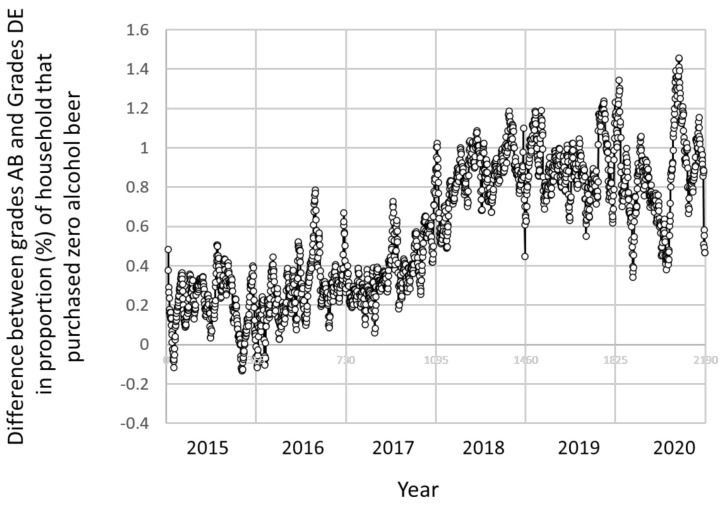
Absolute difference between social grades AB and DE in proportion (%) of households that reported at least one purchase of zero alcohol for any day that a household made an alcohol purchase by study day, 2015 to 2020. Data points: daily.

For purchase of zero alcohol beer, we also examined differences based on the social grade over time (Figure 2). The differences by social grade widened over time, and the absolute difference between social grade groups AB and DE increased by 0.0396% (95% CI = 0.0384 to 0.0408) per every three months over the six years 2015 to 2020.

### 3.2. Survey Data

**Figure 3:**
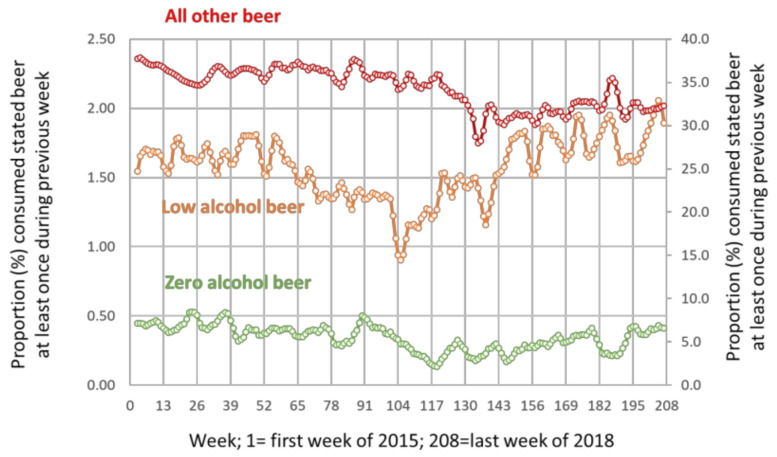
Proportion (%) of survey respondents who reported consuming at least one drink of zero alcohol beer (green, left vertical axis), low alcohol beer (orange, left vertical axis) and all other beer (red, right vertical axis) by week, 2015 to 2018.

Based on the survey data, 0.34% (95% CI = 0.33 to 0.36) of respondents reported consuming at least one drink of zero alcohol beer during the previous week, 1.57% (95% CI = 1.54 to 1.60) of low alcohol beer and 34.3% (95% CI = 34.0 to 34.6) of all other beer. The proportion for all other beer declined for the first two and a half years and then plateaued, Figure 3 (please note, the left vertical axis is for low and zero alcohol beer; and the right vertical axis is for all other beer). For low alcohol beer, there was a decrease during the first two years and then an increase; for zero alcohol beer, it remained low and fairly steady throughout, based on the survey data, 69.2% (95% CI = 67.6 to 70.9) of reported consumption of at least one zero alcohol beer during the previous week took place off-trade (i.e., purchased from supermarkets or off-licences); the proportions for low alcohol beer were 67.8% (95% CI = 67.4 to 68.3); and, for all other beer, 77.2% (95% CI = 76.6 to 77.6).

Table 2 gives the odds ratios (95% confidence intervals) for a respondent consuming at least one zero alcohol beer and at least one low alcohol beer during the previous week by respondent characteristics.

Respondents that were more likely to have consumed zero alcohol beer during the previous week were heavier drinkers of alcohol, consumers of beer with ABV > 3.5%, men, of younger age groups and from ‘higher’ social grades, and, converse to the purchase data, from more deprived residential areas. There was no consistent pattern by region of Great Britain.

Respondents that were more likely to have consumed low alcohol beer during the previous week were heavier drinkers of alcohol, consumers of beer with ABV > 3.5%, men, of younger age groups, from ‘higher’ social grades, and from more deprived residential areas. There was no consistent pattern by region of Great Britain.

## 4. Discussion

Although there were increasing purchases of zero alcohol beer and increasing consumption of low alcohol beer over time, we found relatively few occasions of purchases and consumption of zero and low alcohol beer throughout the time periods surveyed. For every purchase of zero alcohol beer, there were 32 purchases of beer with an ABV of 3.5% or more, and for every purchase of low alcohol beer, there were nearly 14 purchases of beer with an ABV of 3.5% or more. The differences in consumption during the previous week were even higher. For every consumption of at least one zero alcohol beer during the previous week, there were 101 consumptions of at least one beer with an ABV of 3.5% or more, and for every consumption of at least one low alcohol beer, there were nearly 22 consumptions of at least one beer with an ABV of 3.5% or more.

Zero alcohol beer was more likely to be bought and drunk by those who generally bought and drank the most alcohol, those who bought and drank beer with an ABV > 3.5%, men, those with younger ages, and those with higher incomes and higher social grades. There were differences between purchase and consumption by area of residential deprivation. Respondents from less deprived areas (i.e., those with lower incomes, more unemployment, poorer health and poorer housing, etc.) were more likely to purchase zero alcohol beer, but less likely to report recent consumption of zero alcohol beer (i.e., during the previous week). With univariate analyses, those who lived in the north of England were more likely to buy zero alcohol beer, with this difference no longer present in the multivariate analysis. Based on household level purchase data, results suggest the difference between higher and lower social grades increased over time between 2015 and 2020, favouring higher social grades.

Low alcohol beer was more likely to be bought and drunk by those who generally bought and drunk the most alcohol, those who bought and drunk higher strength beer (ABV > 3.5%), men, those with younger ages, those with higher social grades, and those who lived in most deprived residential areas, and with no clear pattern by region of Great Britain.

Thus, although the data were not consistent across all socio-economic measures studied, it seemed that the purchase and consumption of zero and lower alcohol beers was greater amongst younger and more, rather than less, affluent households and respondents, and that the difference favouring the more affluent increased over time. We also found that buying and drinking zero and low alcohol beer was more common amongst those who bought and drunk the most alcohol overall and the most beer, as opposed to those who bought and drank lower amounts of alcohol overall and of beer. Given that we have previously demonstrated that the purchase of zero and lower alcohol beers amongst such heavier buyers of alcohol is associated with buying less grams of alcohol overall, and thus likely health benefit [11], the data might suggest that the promotion of zero and low alcohol beers could lead to increasing health inequalities.

Beyond the qualitative studies mentioned in the introduction [14,15], we are not aware of any published large-scale analyses that have investigated the socio-demographic characteristics of people who drink or purchase zero or low alcohol beer. Our data are consistent with broader behavioural risk factors, for which less healthy behaviours tend to cluster in lower social grades [24] as proposed by Bourdieu [25], with some evidence that innovation towards behaviours that may have less health risk occurs more rapidly amongst higher as opposed to lower social grade groups [26,27]. Zero alcohol beer might be part of purchase and consumption patterns that emerge as a consequence of people having better socioeconomic prospects and related lifestyles, or may be more evident in this group that, on average, buys and drinks more alcohol in Great Britain [28].

Our analyses have several important strengths and limitations. We obtained purchase data from a large number of households and survey data from a large number of respondents. Although relying on compliance at the household level, purchase data based on product bar codes, and verified via digital receipts, is, of itself, objective. The same cannot be said for the survey data which are based on subjective reports of drinking, with such subjective reports tending to underestimate consumption as measured by sales or other recorded data [29]. The timeline follow-back survey method has been criticized for the limited time period of drinking it covers, meaning it potentially misses heavy episodic drinking occasions among participants with a low frequency of such occasions. This limitation for classifying individuals is actually a strength when it comes to the characterization of population averages, however, where the shorter the time period, the smaller the biases due to memory, and the more accurate the population average [30].

Although quality control and compliance for the purchase data are regularly monitored by Kantar, with households only included in the final data set if they adhere to pre-assigned quality control criteria (meeting thresholds for data recording and purchasing volume or spend (based on household size) every four weeks), the data have limitations. Heavy drinkers, particularly male drinkers or those with no fixed address or living in communal establishments, are likely to be under-represented in household panel data [31,32] and that alcohol purchases are under-reported in general in these datasets [33]. For example, compared with the UK Living Costs and Food Survey, KWP households tend to have lower incomes, are more likely to be female headed (as main or primary shoppers), and their expenditure on certain commodity items, including alcohol, tends to be lower [34]. Lower recording of alcohol might reflect the method of recording purchases if not all items purchased are taken home and scanned. Whilst most primary shopping is done by women, secondary top-up shopping, which is more likely done by men, may be less well recorded [35]. Additionally, we are only able to assess changes in off-trade alcohol purchases as opposed to actual levels of alcohol consumption for these time periods. Adults in a household may not have an equal share of the alcohol purchased.

As with all survey-based research on alcohol, data cannot claim representativeness [36]. Representativeness needs to be based on probabilistic sampling design (i.e., all residents need to be assigned a probability >0) combined with high response rates unaffected by systematic non-response [37]. However, these conditions can no longer be reached in modern surveys involving alcohol, no matter which methodology is used [36,38,39]. Instead, post-stratification based on sex, age, social class, and geographical region was used to allow for generalizations to be made for the general population.

The data sets did not include underage drinkers. Finally, the data is just from one jurisdiction (Great Britain) and the findings may not, necessarily, be representative of other jurisdictions.

## 5. Conclusions

Although there is a growing trend [9], the present limited purchase and consumption of zero alcohol and low alcohol beer that our study identified implies that such products could only make a small contribution to efforts to reduce alcohol consumption. Thus, there remains an ongoing need to strengthen the implementation of the WHO SAFER alcohol policy measures to reduce the harm done by alcohol [4].

A key finding from our study is that younger and more socio-economically advantaged households and respondents were more likely to purchase and consume zero alcohol beer in particular. As such, policymakers should be careful in simply prioritising the increased availability of zero and low alcoholic products, as they may be disproportionally beneficial for already better-off populations and could potentially increase health inequalities. Rather, promoting the purchase and consumption of zero and low alcohol products, that may lead to less alcohol consumption [11], needs to go hand in hand with much needed structural policies that help to improve all people’s socioeconomic prospects, aligned with approaches that address the social determinants of health [40,41].

## Figures and Tables

**Table 1:** Analysis at level of each household; odds ratios (OR) (95% confidence intervals) for any purchase of zero alcohol beer (ABV = 0.0%) or low alcohol beer (ABV > 0.0% and ≤3.5%) by household socio-demographic characteristics. In bold: 95% confidence interval does not cross 1.0.

			Zero Alcohol Beer		Low Alcohol Beer	
	Characteristics of Consumers (Please See Methods Section for More Detail)	*n*	Univariate Analysis	Multivariate Analysis	Univariate Analysis	Multivariate Analysis
Total grams of alcohol purchased per week per adult	>70	18,192	1.147 (1.053 to 1.250)	1.132 (1.038 to 1.236)	1.636 (1.538 to 1.741)	1.587 (1.488 to 1.692)
	>21 to ≤70	22,882	1.363 (1.264 to 1.470)	1.217 (1.126 to 1.315)	2.071 (1.960 to 2.189)	1.768 (1.670 to 1.871)
	>7 to ≤21	21,269	0.999 (0.922 to 1.082)	0.908 (0.837 to 0.985)	1.582 (1.495 to 1.675)	1.399 (1.320 to 1.483)
	≤7 (reference category)	17,068	1.000	1.000	1.000	1.000
Purchased any other beer with ABV > 3.5%	Yes	56,503	2.273 (2.090 to 2.472)	2.180 (2.002 to 2.375)	4.775 (4.461 to 5.111)	4.380 (4.089 to 4.692)
	No (reference category)	22,908	1.000	1.000	1.000	1.000
Age group; (age of main shopper, years)	18–34	13,822	1.146 (1.040 to 1.262)	1.102 (0.996 to 1.219)	0.980 (0.916 to 1.049)	0.991 (0.922 to 1.065)
	35–44	18,995	1.438 (1.322 to 1.565)	1.276 (1.167 to 1.394)	1.265 (1.191 to 1.342)	1.191 (1.118 to 1.269)
	45–64	32,426	1.167 (1.082 to 1.260)	1.100 (1.018 to 1.189)	1.318 (1.251 to 1.390)	1.249 (1.183 to 1.319)
	65+ (reference category)	14,168	1.000	1.000	1.000	1.000
Household income group; per adult per household per year, GB pounds	>£22.5 k	19,135	1.556 (1.441 to 1.681)	1.157 (1.059 to 1.265)	0.961 (0.911 to 1.014)	0.858 (0.806 to 0.914)
	>£15–22.5 k	18,252	1.294 (1.194 to 1.403)	1.047 (0.960 to 1.142)	1.063 (1.008 to 1.121)	0.958 (0.903 to 1.016)
	>£8.75–15 k	21,656	1.078 (0.995 to 1.168)	0.989 (0.911 to 1.074)	0.919 (0.872 to 0.968)	0.892 (0.845 to 0.942)
	>£0–8.75 k (reference category)	20,368	1.000	1.000	1.000	1.000
Social grade group	AB (highest)	17,179	1.791 (1.643 to 1.953)	1.526 (1.385 to 1.682)	1.101 (1.039 to 1.167)	1.097 (1.027 to 1.173)
	C1	14,494	1.460 (1.347 to 1.581)	1.323 (1.215 to 1.440)	1.047 (0.994 to 1.102)	1.025 (0.969 to 1.084)
	C2	31,325	1.189 (1.080 to 1.309)	1.095 (0.992 to 1.208)	1.102 (1.038 to 1.171)	1.032 (0.969 to 1.099)
	DE (lowest) (reference category)	16,413	1.000	1.000	1.000	1.000
Area-based residential deprivation group	4 (least deprived)	20,068	1.412 (1.307 to 1.525)	1.233 (1.135 to 1.340)	0.976 (0.925 to 1.029)	0.983 (0.927 to 1.042)
	3	19,689	1.197 (1.105 to 1.296)	1.113 (1.025 to 1.208)	0.988 (0.937 to 1.043)	0.984 (0.930 to 1.041)
	2	20,368	1.082 (0.998 to 1.173)	1.030 (0.948 to 1.119)	0.962 (0.912 to 1.015)	0.988 (0.935 to 1.045)
	1 (most deprived) (reference category)	19,286	1.000	1.000	1.000	1.000
Region of Great Britain	North East	3873	1.235 (1.072 to 1.423)	1.113 (0.961 to 1.289)	0.948 (0.859 to 1.046)	1.012 (0.913 to 1.122)
	North West	8715	1.202 (1.036 to 1.393)	1.127 (0.968 to 1.311)	1.074 (0.969 to 1.190)	1.162 (1.045 to 1.291)
	Yorkshire and the Humber	8023	0.882 (0.737 to 1.056)	.862 (0.718 to 1.034)	0.970 (0.860 to 1.094)	1.021 (0.902 to 1.154)
	West Midlands	7028	1.275 (1.095 to 1.485)	1.251 (1.071 to 1.461)	0.876 (0.786 to 0.977)	0.948 (0.847 to 1.060)
	London	5787	0.987 (0.846 to 1.151)	0.965 (0.825 to 1.128)	1.032 (0.929 to 1.145)	1.121 (1.007 to 1.248)
	East Midlands	7150	1.053 (0.902 to 1.229)	0.997 (0.852 to 1.166)	1.165 (1.049 to 1.294)	1.199 (1.077 to 1.335)
	South West	7710	0.943 (0.800 to 1.112)	0.914 (0.774 to 1.081)	0.714 (0.635 to 0.801)	0.822 (0.729 to 0.926)
	Scotland	6634	0.967 (0.827 to 1.132)	0.931 (0.795 to 1.091)	1.115 (1.004 to 1.239)	1.167 (1.048 to 1.300)
	Wales	4041	1.114 (0.958 to 1.295)	1.068 (0.918 to 1.243)	1.249 (1.128 to 1.383)	1.267 (1.142 to 1.405)
	Eastern	8552	0.916 (0.787 to 1.066)	0.892 (0.766 to 1.040)	1.067 (0.964 to 1.182)	1.085 (0.979 to 1.204)
	South East (reference category)	11,898	1.000	1.000	1.000	1.000

Analyses adjusted for date of first household purchase of alcohol, and length of time (number of days) between first and last purchase of alcohol.

**Table 2:** Analysis at level of each respondent; odds ratios (OR) (95% confidence intervals) for any consumption of zero alcohol beer (ABV = 0.0%) or low alcohol beer (ABV > 0.0% and ≤3.5%) during previous week by respondent socio-demographic characteristics. In bold: 95% confidence interval does not cross 1.0.

			Zero Alcohol Beer		Low Alcohol Beer	
	Characteristics of Consumers (Please See Methods Section for More Detail)	*n*	Univariate Analysis	Multivariate Analysis	Univariate Analysis	Multivariate Analysis
Total grams of alcohol consumed during previous week	>350	7837	2.938 (2.192 to 3.937)	1.654 (1.210 to 2.260)	6.220 (5.454 to 7.093)	3.471 (3.012 to 4.000)
	>200 to ≤350	9851	2.296 (1.710 to 3.082)	1.400 (1.028 to 1.908)	3.885 (3.377 to 4.469)	2.312 (1.994 to 2.681)
	>100 to ≤200	16,534	1.670 (1.271 to 2.195)	1.061 (0.795 to 1.416)	2.686 (2.353 to 3.067)	1.657 (1.440 to 1.906)
	≤100 (reference category)	70,413	1.000	1.000	1.000	1.000
Consumed any other beer with ABV > 3.5%	Yes	35,864	3.132 (2.540 to 3.862)	2.479 (1.940 to 3.167)	4.633 (4.164 to 5.156)	3.017 (2.664 to 3.417)
	No (reference category)	68,771	1.000	1.000	1.000	1.000
Gender	Men	53,409	1.759 (1.422 to 2.175)	1.542 (1.218 to 1.952)	1.946 (1.754 to 2.158)	1.366 (1.216 to 1.535)
	Women (reference category)	51,226	1.000	1.000	1.000	1.000
Age group, years	18–34	43,426	3.845 (2.381 to 6.209)	3.935 (2.406 to 6.437)	3.340 (2.655 to 4.201)	3.713 (2.927 to 4.709)
	35–44	18,718	2.160 (1.274 to 3.663)	1.866 (1.091 to 3.192)	2.854 (2.235 to 3.645)	2.518 (1.960 to 3.236)
	45–64	30,134	1.223 (0.717 to 2.086)	1.196 (0.700 to 2.044)	1.487 (1.160 to 1.907)	1.398 (1.088 to 1.796)
	65+ (reference category)	12,357	1.000	1.000	1.000	1.000
Social grade group	AB (highest)	26,202	1.894 (1.432 to 2.504)	1.352 (1.014 to 1.803)	1.392 (1.214 to 1.597)	0.961 (0.833 to 1.109)
	C1	22,147	1.745 (1.300 to 2.344)	1.140 (0.839 to 1.551)	1.530 (1.331 to 1.759)	1.017 (0.877 to 1.179)
	C2	24,538	1.094 (0.792 to 1.512)	0.986 (0.710 to 1.369)	1.281 (1.111 to 1.476)	1.054 (0.910 to 1.221)
	DE (lowest) (reference category)	31,748	1.000	1.000	1.000	1.000
Deprivation group	4 (least deprived)	23,325	0.713 (0.534 to 0.953)	0.764 (0.563 to 1.038)	0.827 (0.723 to 0.946)	0.903 (0.782 to 1.044)
	3	23,739	0.661 (0.492 to 0.889)	0.722 (0.531 to 0.981)	0.742 (0.646 to 0.852)	0.832 (0.719 to 0.962)
	2	27,192	0.926 (0.716 to 1.197)	0.950 (0.728 to 1.238)	0.812 (0.714 to 0.924)	0.862 (0.753 to 0.986)
	1 (most deprived) (reference category)	30,379	1.000	1.000	1.000	1.000
Region of Great Britain	North East	4474	0.606 (0.333 to 1.103)	0.756 (0.410 to 1.393)	1.220 (0.891 to 1.670)	1.574 (1.142 to 2.170)
	North West	11,292	0.783 (0.431 to 1.421)	0.950 (0.519 to 1.740)	1.133 (0.818 to 1.570)	1.465 (1.051 to 2.042)
	Yorkshire and the Humber	10,185	0.698 (0.339 to 1.439)	0.789 (0.381 to 1.635)	0.990 (0.675 to 1.453)	1.086 (0.737 to 1.601)
	West Midlands	9544	0.902 (0.516 to 1.575)	1.071 (0.609 to 1.885)	1.338 0(0.982 to 1.822)	1.641 (1.198 to 2.247)
	London	10,207	0.669 (0.357 to 1.253)	0.820 (0.435 to 1.546)	0.932 (0.663 to 1.312)	1.170 (0.827 to 1.654)
	East Midlands	8961	1.052 (0.590 to 1.874)	1.135 (0.632 to 2.037)	1.371 (0.993 to 1.894)	1.571 (1.132 to 2.181)
	South West	9031	2.208 (1.310 to 3.721)	1.899 (1.122 to 3.214)	2.370 (1.754 to 3.203)	2.142 (1.579 to 2.907)
	Scotland	13,383	0.911 (0.507 to 1.637)	0.912 (0.507 to 1.641)	1.609 (1.175 to 2.203)	1.708 (1.244 to 2.346)
	Wales	4880	0.666 (0.361 to 1.230)	0.677 (0.366 to 1.251)	1.337 (0.972 to 1.839)	1.400 (1.015 to 1.931)
	Eastern	10,077	0.836 (0.469 to 1.490)	0.829 (0.465 to 1.480)	1.431 (1.047 to 1.956)	1.465 (1.069 to 2.008)
	South East (reference category)	12,601	1.000	1.000	1.000	1.000

Analyses adjusted for date of completion of survey.

## Data Availability

Kantar Worldpanel data cannot be shared due to licensing restrictions.

## References

[1] Wood A., Kaptoge S., Butterworth A., Paul D., Burgess S., Sweeting M., Bell S., Astle W., Willeit P., Bolton T. (2018). Risk thresholds for alcohol consumption: Combined analysis of individual-participant data for 599,912 current drinkers in 83 prospective studies. Lancet.

[2] World Health Organization (WHO) (2018). Global Status Report on Alcohol and Health.

[3] World Health Organization (2013). Global Action Plan for the Prevention and Control of NCDs 2013–2020.

[4] World Health Organization (WHO) (2020). SAFER, Alcohol Control Initiative. https://www.who.int/substance_abuse/safer/en/.

[5] World Health Organization (2010). Global Strategy to Reduce the Harmful Use of Alcohol.

[6] Rehm J., Lachenmeier D.W., Jané-Llopis E., Imtiaz S., Anderson P. (2016). On the evidence base of reducing ethanol content in beverages to reduce the harmful use of alcohol. Lancet Gastroenterol. Hepatol..

[7] Capitello R., Todirica I.C., Capitello R., Maehle N. (2021). Understanding the Behavior of Beer Consumers.

[8] Salanță L.C., Coldea T.E., Ignat M.V., Pop C.R., Tofană M., Mudura E., Borșa A., Pasqualone A., Zhao H. (2020). Non-Alcoholic and Craft Beer Production and Challenges. Processes.

[9] Corfe S., Hyde R., Shepherd J. Alcohol-Free and Low-Strength Drinks Understanding Their Role in Reducing Alcohol-Related Harms. https://www.smf.co.uk/publications/no-low-alcohol-harms/.

[10] UK Government (2019). Advancing our Health: Prevention in the 2020s—Consultation Document. https://www.gov.uk/government/consultations/advancing-our-health-prevention-in-the-2020s/advancing-our-health-prevention-in-the-2020s-consultation-document.

[11] Anderson P., Llopis E.J., O’Donnell A., Manthey J., Rehm J. (2020). Impact of low and no alcohol beers on purchases of alcohol: Interrupted time series analysis of British household shopping data, 2015–2018. BMJ Open.

[12] Hagemann M.H., Bogner K., Marchioni E., Braun S. (2016). Chances for dry-hopped non-alcoholic beverages? Part 1: Concept and market prospects. Brew. Sci..

[13] Hagemann M.H., Schmidt-Cotta V., Marchioni E., Braun S. (2017). Chance for Dry-hopped Non-alcoholic Beverages? Part 2: Health Properties and Target Consumers. Brew. Sci..

[14] Chrysochou P. (2014). Drink to get drunk or stay healthy? Exploring consumers’ perceptions, motives and preferences for light beer. Food Qual. Prefer..

[15] Silva A.P., Jager G., van Bommel R., van Zyl H., Voss H.-P., Hogg T., Pintado M.M., de Graaf C. (2016). Functional or emotional? How Dutch and Portuguese conceptualise beer, wine and non-alcoholic beer consumption. Food Qual. Prefer..

[16] (2018). The Alcohol Change Report. https://alcoholchange.org.uk/get-involved/campaigns/the-alcohol-change-report.

[17] Leicester A., Oldfield Z., Oldfield Z. (2009). Using Scanner Technology to Collect Expenditure Data*. Fisc. Stud..

[18] Jakobsen J.C., Gluud C., Wetterslev J., Winkel P. (2017). When and how should multiple imputation be used for handling missing data in randomised clinical trials—A practical guide with flowcharts. BMC Med. Res. Methodol..

[19] National Readership Survey (2019). Social Class London: National Readership Survey. http://www.nrs.co.uk/nrs-print/lifestyle-and-classification-data/social-grade/.

[20] GOV.UK National Statistics: English Indices of Deprivation 2019. https://www.gov.uk/government/statistics/english-indices-of-deprivation-2019.

[21] Gov.scot (2020). Scottish Index of Multiple Deprivation (SIMD) 2020 Technical Notes. https://www.gov.scot/publications/simd-2020-technical-notes/.

[22] Gov.Wales (2019). Welsh Index of Multiple Deprivation (full Index update with ranks). https://gov.wales/welsh-index-multiple-deprivation-full-index-update-ranks-2019.

[23] IBM Corp (2019). IBM SPSS Statistics for Windows, Version 26.0..

[24] Anderson P., Llopis E.J., O’Donnell A., Kaner E. (2021). Impact of COVID-19 Confinement on Alcohol Purchases in Great Britain: Controlled Interrupted Time-Series Analysis During the First Half of 2020 Compared With 2015–2018. Alcohol Alcohol..

[25] Tomlinson M. (2003). Lifestyle and Social Class European Sociological Review. Eur. Sociol. Rev..

[26] Bourdieu P. (1984). Distinction: A Social Critique of the Judgement of Taste.

[27] Petrovic D., de Mestral C., Bochud M., Bartley M., Kivimaki M., Vineis P., Mackenbach J., Stringhini S. (2018). The contribution of health behaviors to socioeconomic inequalities in health: A systematic review. Prev. Med..

[28] Pampel F.C., Krueger P.M., Denney J.T. (2010). Socioeconomic Disparities in Health Behaviors. Annu. Rev. Sociol..

[29] Office for National Statistics (2018). Adults drinking habits in Great Britain: 2017. https://www.ons.gov.uk/peoplepopulationandcommunity/healthandsocialcare/drugusealcoholandsmoking/datasets/adultdrinkinghabits.

[30] Kilian C., Manthey J., Probst C., Brunborg G.S., Bye E.K., Ekholm O., Kraus L., Moskalewicz J., Sieroslawski J., Rehm J. (2020). Why Is Per Capita Consumption Underestimated in Alcohol Surveys? Results from 39 Surveys in 23 European Countries. Alcohol Alcohol..

[31] Stockwell T., Zhao J., Chikritzhs T., Greenfield T.K. (2008). What did you drink yesterday? Public health relevance of a recent recall method used in the 2004 Australian National Drug Strategy Household Survey. Addiction.

[32] Gill J., Black H., Rush R., O’May F., Chick J. (2017). Heavy Drinkers and the Potential Impact of Minimum Unit Pricing—No Single or Simple Effect?. Alcohol Alcohol..

[33] Gorman E., Leyland A., McCartney G., White I., Katikireddi S., Rutherford L., Graham L., Gray L. (2014). Assessing the Representativeness of Population-Sampled Health Surveys Through Linkage to Administrative Data on Alcohol-Related Outcomes. Am. J. Epidemiol..

[34] Pechey R., Jebb S.A., Kelly M.P., Almiron-Roig E., Conde S., Nakamura R., Shemilt I., Suhrcke M., Marteau T.M. (2013). Socioeconomic differences in purchases of more vs. less healthy foods and beverages: Analysis of over 25,000 British households in 2010. Soc. Sci. Med..

[35] Leicester A. (2012). How might in-home scanner technology be used in budget surveys?. How Might In-Home Scanner Technology Be Used in Budget Surveys?.

[36] Rehm J., Kilian C., Rovira P., Shield K.D., Manthey J. (2021). The elusiveness of representativeness in general population surveys for alcohol. Drug Alcohol Rev..

[37] Kruskal W., Mosteller F. (1979). Representative Sampling, III: The Current Statistical Literature. Int. Stat. Rev..

[38] Stedman R.C., Connelly N.A., Heberlein T.A., Decker D.J., Allred S.B. (2019). The end of the (research) world as we know it? Understand-ing and coping with declining response rates to mail surveys. Soc. Nat. Resour..

[39] Habermann H., Kennedy C., Lahiri P. (2017). A Conversation with Robert Groves. Stat. Sci..

[40] Marmot. M., Wilkinson R. (2005). Social Determinants of Health.

[41] Marmot M., Bell R. (2019). Social determinants and non-communicable diseases: Time for integrated action. BMJ.

